# Neuroprotective Effect of Polyphenol Extracts from *Terminalia chebula* Retz. against Cerebral Ischemia-Reperfusion Injury

**DOI:** 10.3390/molecules27196449

**Published:** 2022-09-30

**Authors:** Kuan Lin, Mei Zhou, Changlong Leng, Xiaoqing Tao, Rong Zhou, Youwei Li, Binlian Sun, Xiji Shu, Wei Liu

**Affiliations:** Wuhan Institutes of Biomedical Sciences, School of Medicine, Jianghan University, Wuhan 430056, China

**Keywords:** neuroprotective effects, *Terminalia chebula*, oxygen-glucose deprivation/re-oxygenation, ischemia-reperfusion, apoptosis, oxidative stress

## Abstract

Current therapies for ischemic stroke are insufficient due to the lack of specific drugs. This study aimed to investigate the protective activity of polyphenol extracts from *Terminalia chebula* against cerebral ischemia-reperfusion induced damage. Polyphenols of ethyl acetate and n-butanol fractions were extracted from *T. chebula*. BV2 microglial cells exposed to oxygen-glucose deprivation/reoxygenation and mice subjected to middle cerebral artery occlusion/reperfusion were treated by TPE and TPB. Cell viability, cell morphology, apoptosis, mitochondrial membrane potential, enzyme activity and signaling pathway related to oxidative stress were observed. We found that TPE and TPB showed strong antioxidant activity in vitro. The protective effects of TPE and TPB on cerebral ischemia-reperfusion injury were demonstrated by enhanced antioxidant enzyme activities, elevated level of the nucleus transportation of nuclear factor erythroid 2-related factor 2 and expressions of antioxidant proteins, with a simultaneous reduction in cell apoptosis and reactive oxygen species level. In conclusion, TPE and TPB exert neuroprotective effects by stimulating the Nrf2 signaling pathway, thereby inhibiting apoptosis.

## 1. Introduction

Ischemic stroke is a cerebrovascular disease of the central nervous system and causes death and physical disability [1]. Numerous studies have shown that microglia are the main immune defense cells in the brain for resisting invasion and damage [2]. Furthermore, excessive microglial activation is considered to affect neurotoxicity in ischemic stroke by releasing excessive reactive oxygen species (ROS) and inflammatory cytokines [3,4,5]. Reperfusion after oxygen and glucose deprivation causes more serious brain damage by ROS overproduction, calcium overload, and blood-brain barrier disruption [6,7,8]. These pathophysiological changes are involved in regulating mitochondrial function, oxidative stress, inflammation, and cell apoptosis in ischemic stroke. Finally, oxidative stress is recognized as a major factor causing the brain damage [9,10].

Mitochondrial membrane potential (MMP) is crucial for maintaining mitochondrial function; furthermore, the mitochondrial morphology changes from integrity to small fragments after cerebral ischemia/reperfusion injury, and fragmented mitochondria induces MMP changes to a lower potential [11]. These fragments may cause mitochondrial dysfunction, and therefore, induce oxidative stress and excessive ROS release [12]. Subsequently, these biochemical changes cause the release of pro-apoptotic factors from mitochondria into the cytosol [13]. Therefore, these biochemical substances induce the activation of mitochondria-dependent apoptosis [14,15].

The nuclear transcription Nrf2 is a key molecule that mitigates the effects of oxidative stress in cells [16]. Translocation of Nrf2 from cytosol to nucleus promotes the expression of downstream antioxidant genes, including heme oxygenase-1 (HO-1), and enhances the activity of antioxidant-related enzymes, including superoxide dismutase (SOD) and glutathione peroxidase (GSH), to scavenge excessive ROS [17,18]. However, oxygen and glucose deprivation in ischemic stroke results in excessive ROS, and then Nrf2 is activated and released from keap1 into the nucleus to bind to antioxidant response elements (ARE) in order to regulate the expression levels of several antioxidant enzymes; on the other hand, Nrf2-downstream proteins can mitigate the deleterious effects of oxidants produced in excess during reoxygenation [16,19].

*Terminalia chebula* is a member of the Combretaceae family and found often in tropical and subtropical Asia; the shape of the dried fruit is long and oval, somewhat like an olive and dark in appearance [20]. In China, the dried fruit of *T. chebula* is widely used to soak in water or grind into granules to drink for treating various chronic inflammations. Furthermore, *T.* *chebula* extracts or its components have demonstrated antioxidative, anti-inflammatory [21], neuroprotective [22], hepatoprotective [23], antibacterial [24], antidiabetic and gastroprotective activity in pharmacological studies [25]. Recent research has reported that the major phenolic compounds of *T. chebula* are gallic acid, ellagic acid, corilagin, chebulinic acid and chebulagic acid [26]. *T. chebula* extract has potential neuroprotective activity against neuronal damage in an experimental ischemic stroke model [27] and Aβ aggregation induced toxicity [28], and to improve memory impairment [29]. However, the neuroprotective effect of *T. chebula* extract on the brain injury caused by ischemic stroke and the molecular mechanism were not understood.

This research assessed the neuroprotective activity of polyphenol extracts from *T. chebula* against OGD/R injury in BV2 microglial cells and middle cerebral artery occlusion (MCAO) injury in mice. Firstly, polyphenols from the ethyl acetate fraction from *T. chebula* methanol extract (TPE) and n-butanol fraction (TPB) were isolated and collected. Then, the OGD/R model in vitro and MACO mice model were established to determine the protective effects of the extracts. We demonstrated that the treatment with TPE and TPB attenuated cell apoptosis and oxidative stress, which indicates its potential application in the prevention or treatment of ischemic stroke.

## 2. Results

### 2.1. Different Fractions from T. chebula Extract and Antioxidant Activities

**Figure 1:**
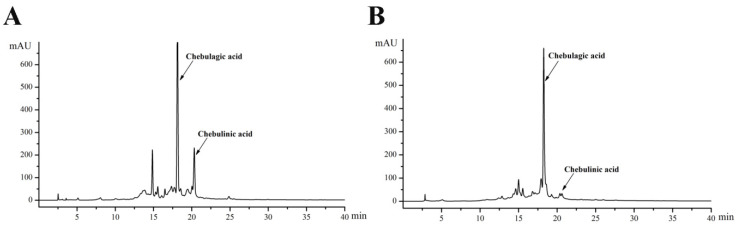
HPLC chromatograms of polyphenols from *T. chebula* extract, (**A**) ethyl acetate and (**B**) n-butanol fractions.

Previous study has shown the highest polyphenol content was observed in the 50% methanol extract of *T. chebula*. The primary bioactive phenolic acids of TPE (Figure 1A) and TPB (Figure 1B) were identified through HPLC after fractionation and purification of the crude extract. Based on the corresponding time of the liquid chromatography peak for the standard substance, the major components were identified as chebulagic acid (18.178 min) and chebulinic acid (20.239 min). Quantitative analysis of the two components suggested that chebulinic and chebulagic acid levels in TPE (92.9 mg/g and 246.2 mg/g, respectively) were significantly higher than those in TPB (10.6 mg/g and 195.7 mg/g, respectively). 

As shown in Table 1, antioxidant activities of TPE and TPB were determined through DPPH•, ABTS•+, and OH• scavenging experiments. The DPPH• scavenging abilities of TPE and TPB were equivalent and stronger than those of vitamin C. Additionally, the ABTS-free radical scavenging ability of TPE was strongest among the three components; furthermore, TPE and TPB had a better ability to scavenge hydroxyl radicals compared with vitamin C. Compared with TPB and vitamin C, TPE had stronger antioxidant activity in vitro for scavenging DPPH, ABTS and hydroxyl radicals.

### 2.2. Effects of TPE and TPB on the Viability of Cells

**Figure 2:**
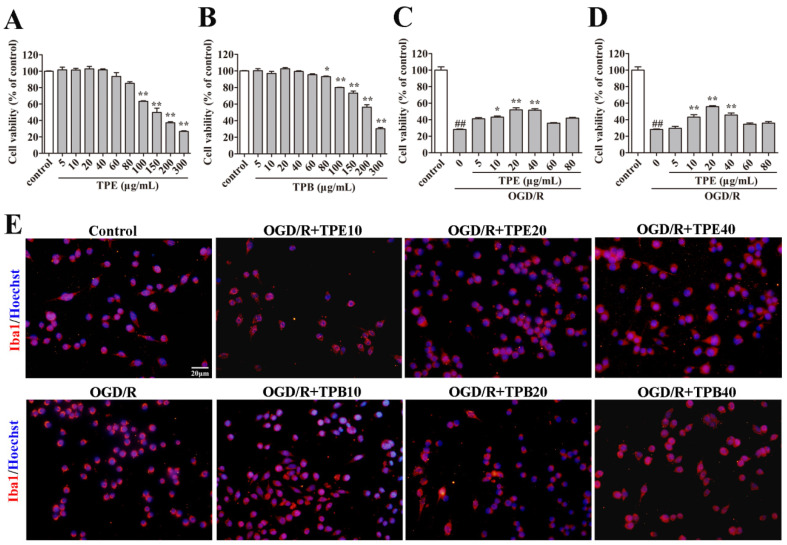
Effects of TPE and TPB on BV2 microglial cells. (**A**,**B**) Cell toxicities of TPE and TPB. (**C**,**D**) The protective effects of TPE and TPB against OGD/R. (**E**) Morphological examination of BV2 microglial cells stained using Iba1 and Hoechst under a fluorescence microscope. ## *p* < 0.01 vs. control group; * *p* < 0.05, ** *p* < 0.01 vs. OGD/R group. OGD/R: oxygen-glucose deprivation/reoxygenation.

To clarify the biological safety of TPB and TPE, we used CCK8 test in BV2 microglia cells. TPE and TPB displayed less significant cytotoxic effects on cells at the concentrations of 1–80 μg/mL; however, cytotoxicity increased with increasing concentration (Figure 2A,B). Therefore, we used 0–80 μg/mL TPE and TPB for subsequent cytoprotection studies in OGD/R treated BV2 microglial cells. We found that TPE and TPB at concentrations of 10, 20, and 40 μg/m L had significant protective effects against OGD/R-induced damage in BV2 microglial cells (Figure 2C,D). On the other hand, we performed immunofluorescence experiments by Iba1 and Hoechst staining to evaluate protective effects of TPB and TPE in OGD/R treated BV2 microglial cells. The morphology of BV2 microglial cells turned into a shrunken circle with fewer extending tentacles after OGD/R treatment; contrastingly, the cell tentacles were recovered in the TPE and TPB treatment groups (Figure 2E). We found that 10, 20, and 40 μg/mL of TPE and TPB treatments had remarkable protective effects on BV2 microglial cells against OGD/R-induced structural damage. Therefore, we used doses of 10, 20, and 40 μg/mL as the treatment concentrations of TPE and TPB in OGD/R for the subsequent experiments to study the effects and mechanisms of cytoprotective.

### 2.3. TPE and TPB Ameliorate OGD/R Injury by Reducing Cell Apoptosis

**Figure 3:**
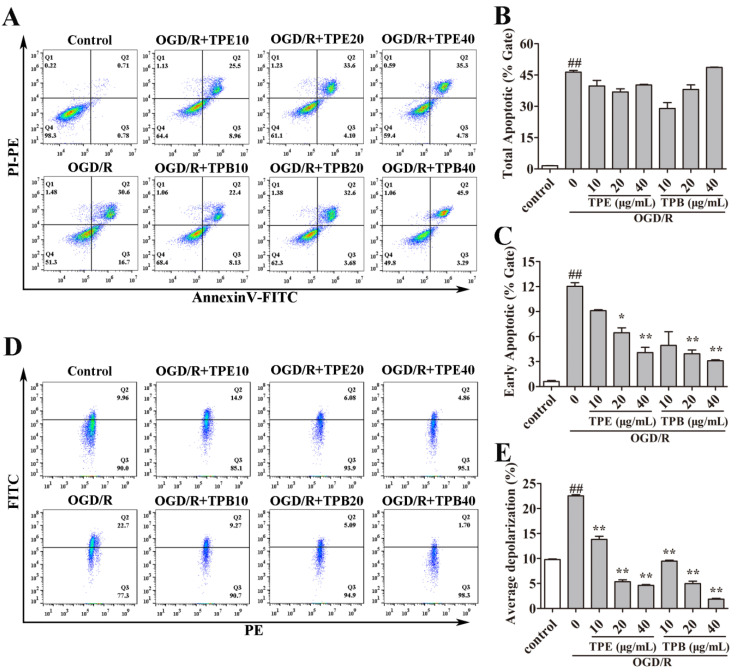
TPE and TPB ameliorated OGD/R injury by reducing early cell apoptosis and maintaining mitochondrial membrane potential. (**A**) Flow cytometer images of cell apoptosis are shown. (**B**) We calculated the total apoptotic ratio by measuring FITC and PI fluorescence. (**C**) Quantification of early apoptotic ratio is shown. (**D**) Flow cytometer images of MMP are displayed. (**E**) Quantification results of JC-1 monomers (green fluorescence) compared with JC-1 aggregates (red fluorescence) labeled using JC-1 probe are shown. ## *p* < 0.01 vs. control group; * *p* < 0.05, ** *p* < 0.01 vs. OGD/R group.

Apoptosis is among the major pathways that cause cell death after OGD/R in cells [30]. To study whether the cytoprotective effect of TPB and TPE was associated with apoptosis, flow cytometry of Annexin V-FITC/PI double staining was performed. The total apoptotic ratio reached 46.37% ± 1.37% after OGD/R (Figure 3A), indicating that OGD/R could promote cell apoptosis. Moreover, the treatment groups of TPE and TPB showed no significant differences in the percentages of total apoptotic cells compared with the OGD/R group. This suggested that TPE and TPB treatments did not alter the total apoptotic ratio (Figure 3B). For more detail, the early apoptotic ratio was quantified. We found that the OGD/R-induced early apoptotic ratio of BV2 microglial cells was significantly reduced after treating with 20 μg/ mL and 40 μg/mL TPE or TPB (Figure 3C).

Early apoptosis was associated with mitochondrial dysfunction [31]. Further, we assessed the varieties of MMP using JC-1 after TPE and TPB treatments. MMP is a primary indicator of mitochondrial function, with its loss being correlated with mitochondrial dysfunction and causing early cell apoptosis [31]. Normal cells mainly exhibited red fluorescence and weak green fluorescence. Consistent with results of flow cytometry, OGD/R-injured BV2 microglial cells showed a markedly enhanced ratio of green fluorescence compared with the control, indicating the occurrence of mitochondrial membrane depolarization. Treatment with TPE and TPB significantly ameliorated OGD/R-induced MMP dissipation. Taken together, our findings showed that TPE and TPB had positive effects on OGD/R damage by ameliorating early cell apoptosis and maintaining MMP (Figure 3D).

### 2.4. Enzyme Activities Related to Oxidative Stress

**Figure 4:**
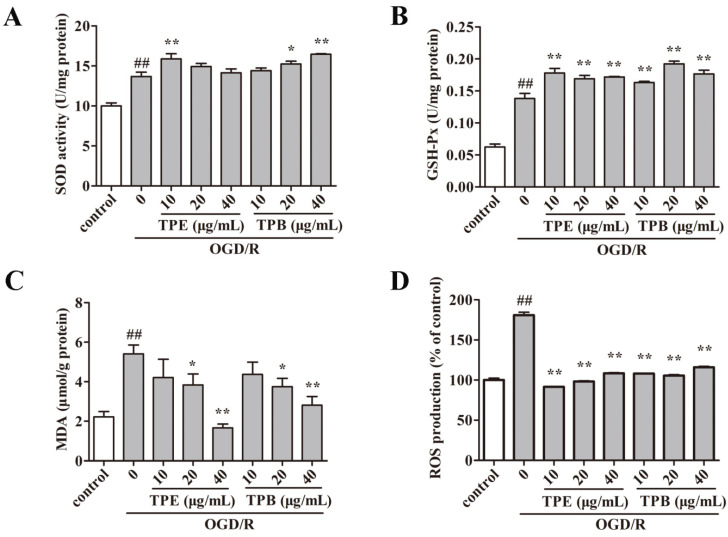
TPE and TPB effects on OGD/R injury was measured by quantifying SOD activity (**A**), MDA levels (**B**), GSH-Px levels (**C**) and ROS production (**D**). ## *p* < 0.01 vs. control group, * *p* < 0.05, ** *p* < 0.01 vs. OGD/R group.

In vitro tests confirmed that TPB and TPE have anti-oxidative stress effects, so we assessed SOD activity (Figure 4A) and GSH-P x activity (Figure 4B) to study the effect of TPE and TPB against OGD/R injury. There were increased SOD and GSH-P x activities after OGD/R compared with the control group (*p* < 0.01), which was indicative of OGD/R-induced antioxidant enzyme activities. Additionally, the GSH-P x activity was significantly increased (*p* < 0.01) in the TPE and TPB treatment groups. Moreover, SOD activity was improved by 10 μ g/mL TPE, as well as by 20 and 40 μg/mL TPB. MDA is produced under oxidative stress as a by-product of membrane lipid peroxidation and induces damage of the plasma membrane. Levels of MDA were markedly increased after OGD/R (*p* < 0.01). TPE (20 and 40 μg/mL) and TPB (20 and 40 μg/mL) treatment significantly reduced MDA levels (Figure 4C). These results suggested that TPE and TPB could alleviate the reduction of antioxidant enzyme activity after OGD/R. As a major factor for regulating cellular oxidative stress, ROS overproduction after OGD/R in BV2 microglial cells was markedly mitigated by TPE and TPB treatments (Figure 4D). These findings indicated that TPE and TPB treatment restored OGD/R-induced oxidative damage in BV2 microglial cells.

### 2.5. TPE and TPB Promotes the Nuclear Translocation of Nrf2 in BV2 Cells

**Figure 5:**
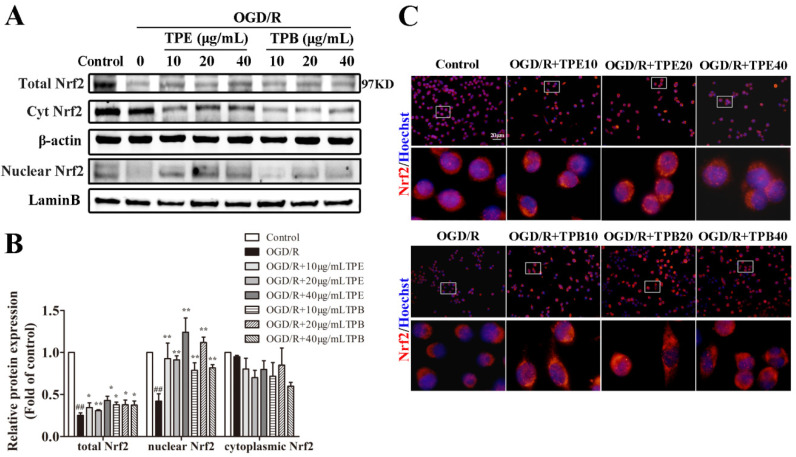
The Nrf2 signaling pathway was stimulated by treatments with TPE and TPB in OGD/R-injured BV2 microglial cells. (**A**) TPE and TPB treatments prompted the nucleus transportation of Nrf2 after OGD/R. (**B**) Relative protein values were normalized to those of the control group using a histogram. (**C**) Representative photomicrographs of BV2 microglial cells stained using Nrf2 and Hoechst as observed under a fluorescence microscope are shown. ## *p* < 0.01 vs. control group, * *p* < 0.05, ** *p* < 0.01 vs. OGD/R group.

Nrf2 signaling pathway is a classical pathway regulating oxidative stress injury. To determine whether TPE and TPB increase anti-oxidative factors by stimulating the Nrf2 signaling pathway, we separated nuclear and cytoplasmic proteins followed by western blotting to analyze Nrf2 protein expression. The OGD/R group showed significantly lower total and nuclear levels of Nrf2 (*p* < 0.01) compared with the normoxic condition. Notably, TPE and TPB remarkably increased Nrf2 protein expression in the nucleus (Figure 5A,B). Our findings suggested that the protective effects of TPE and TPB involved stimulation of Nrf2 expression in the nucleus. Further, we examined whether TPE and TPB could promote the nuclear translocation of Nrf2; cellular immunofluorescence staining was applied to demonstrate the subcellular location of Nrf2 in BV2 microglial cells. Photomicrographs revealed smaller overlap areas of red and blue fluorescence in the OGD/R group, which was enhanced after TPE and TPB treatments (Figure 5C). OGD exposure decreased the nuclear localization of Nrf2; furthermore, treatment with TPE and TPB promoted the nuclear localization of Nrf2. Consistent with the western blotting results, these findings suggested that TPE and TPB facilitated the translocation of Nrf2 to nucleus and enhanced Nrf2 expression in nucleus.

### 2.6. TPE and TPB Stimulate the Nrf2/HO-1 Signaling Pathway in BV2 Cells

**Figure 6:**
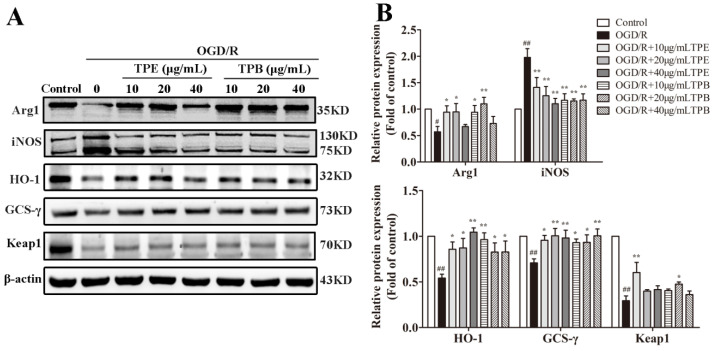
OGD/R-injured cells were treated with TPE or TPB. (**A**) TPE and TPB concentrations on the expression of Arg1, iNOS, HO-1, GCS-γ, Keap1. (**B**) Relative protein values were normalized to those of the control group using a histogram. # *p* < 0.05, ## *p* < 0.01 vs. control group, * *p* < 0.05, ** *p* < 0.01 vs. OGD/R group.

Nrf2 binds to Keap1 in the cytoplasm under physiological conditions; however, excessive oxygen radicals promote Keap1 separation from Nrf2 after oxygen and glucose deprivation. Additionally, activated Nrf2 is transferred from cytoplasm to the nucleus, followed by the initiation of transcription of the downstream antioxidant gene HO-1. Therefore, Nrf2/HO-1 is an endogenous intracellular antioxidant system [32]. Arg1 is a typical marker for M2 macrophage/microglia activation that participates in arginine metabolism. Excessive oxidative stress promotes iNOS expression and exerts its effect by producing nitric oxide. OGD/R-induced damage remarkably decreased Arg1 and antioxidant protein expression (*p* < 0.01) but increased iNOS expression (*p* < 0.01). Treatments with TPE and TPB (10, 20 and 40 µg/mL) significantly increased HO-1 and GCS-γ expression (all *p* < 0.05), and simultaneously decreased iNOS protein expression (*p* < 0.01). Moreover, treatment with TPE (40 μg/mL) and TPB (20 μg/mL) increased Keap1 protein expression (*p* < 0.05) (Figure 6A,B). These results are consistent with previous findings regarding antioxidant enzyme activities. Our findings suggested that TPE and TPB reduce OGD/R damage by adjusting the expression of related anti-oxidative proteins.

### 2.7. TPE and TPB Improve Enzyme Activities Related to Oxidative Stress in MCAO Mice

**Figure 7:**
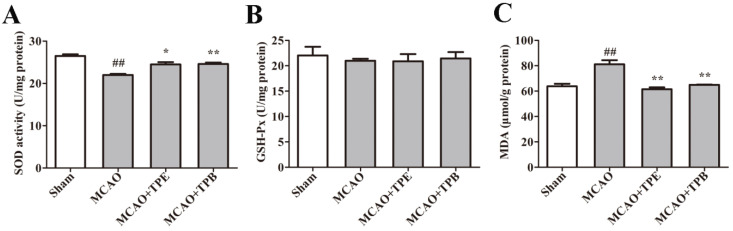
TPE and TPB protection of the brain from cerebral ischemia/reperfusion injury was measured by quantifying SOD activity (**A**), GSH-P x levels (**B**) and MDA levels (**C**). ## *p* < 0.01 vs. sham group, * *p* < 0.05, ** *p* < 0.01 vs. MCAO group.

To further study whether TPE and TPB can protect the brain against cerebral ischemia-reperfusion injury, a MCAO mouse model was used. Primarily, SOD activity (Figure 7A) and GSH-P x activity (Figure 7B) were quantitated to assess the effects of TPE and TPB. We found there were a significantly decreased SOD activity in MCAO mice compared with the sham group (*p* < 0.01), which was indicative of MCAO injury suppressed antioxidant enzyme activities. Moreover, SOD activity was significantly increased in the TPE (*p* < 0.05) and TPB (*p* < 0.01) treatment groups. However, there was no statistical difference in groups of GSH-P x activity. We next investigated the levels of MDA, produced under oxidative stress, which were markedly increased in the MCAO group (*p* < 0.01). However, treatments of TPE and TPB significantly inhibited MDA production (Figure 7C). These results collectively indicated that TPE and TPB could alleviate the reduction of antioxidant enzyme activity in cerebral ischemia-reperfusion injury.

### 2.8. TPE and TPB Protect Brain from MCAO Injury by Reducing Apoptosis and Stimulating the Nrf2/HO-1 Signaling Pathway

**Figure 8:**
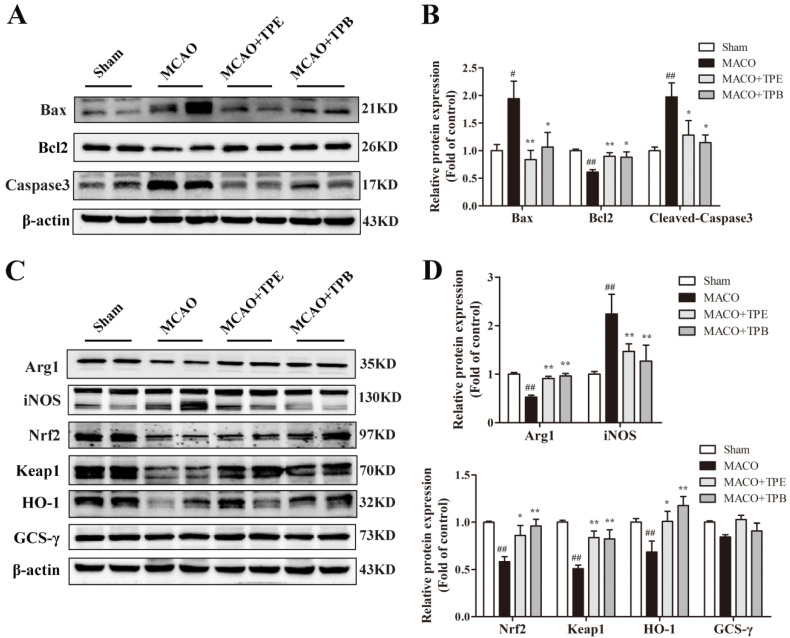
TPE and TPB reduced the cell apoptosis after cerebral ischemia/reperfusion injury by stimulating the Nrf2/HO-1 signaling pathway. (**A**) Effects of TPE and TPB on the expression of Bax, Bcl2, Caspase 3. (**C**) Effects of TPE and TPB on the expression of Arg1, iNOS, Nrf2, Keap1 HO-1, GCS-γ. (**B**,**D**) Relative protein values were normalized to those of sham group using a histogram. # *p* < 0.05, ## *p* < 0.01 vs. sham group; * *p* < 0.05, ** *p* < 0.01 vs. MCAO group.

To investigate whether TPE and TPB have the same protective role in MCAO mice, western blot was used to analyse several apoptosis proteins in cerebral cortex including Bax, Bcl2 and Caspase3. The result showed that following MCAO injury, the mice had increased expression of Bax and Caspase3 and decreased expression of Bcl2. In addition, TPE and TPB treatment mice showed lower Bax and Caspase3 expression and higher Bcl2 expression than MCAO mice (Figure 8A,B). Because TPE and TPB had improved excellent antioxidant activity in vitro, to provide further mechanisms for the protective effects of TPE and TPB on cerebral ischemia-reperfusion injury, we tested the Nrf2/HO-1 signaling pathway, which was well-known related to oxidative stress. After model induction, the activation of iNOS inflammation and the inhibition of the Nrf2/HO-1 signaling pathway in the brain were observed. The protein level of iNOS was markedly increased in MCAO mice, which was prevented by TPE and TPB; in contrast, Arg1 was markedly decreased in MCAO mice but reversed by TPE and TPB treatments (Figure 8C,D). Furthermore, the MCAO mice showed significantly lower total level of Nrf2 (*p* < 0.01) compared with the sham mice. TPE and TPB treatment mice had a high expression of Nrf2, which is extensively involved in ameliorating cerebral ischemia-reperfusion injury (Figure 8C,D). In related analyses, we found that MCAO reduced the expression of the downstream proteins Keap1 and HO-1 (*p* < 0.01), while TPE and TPB treatments enhanced the expression of these antioxidant proteins; however, there were no significant differences between MCAO and TPE and TPB groups for GCS-γ (Figure 8C,D). The above results suggested that TPE and TPB exerted protective effects in MCAO mice by inhibiting apoptosis and stimulating the Nrf2/HO-1 signaling pathway. 

## 3. Discussion

Microglias are the primary immune cells and involved in the development of neurological diseases. The microglial cellular processes are highly dynamic in the healthy brain with a rapid response to injury. They are reactive to brain injury induced by ischemic stroke and actively produce signaling molecules for facilitating homeostasis [4]. OGD/R is a classic cellular model of cerebral ischemia/reperfusion injury. This study evaluated the neuroprotective effects of TPE and TPB against OGD/R-induced damage in BV2 microglial cells and cerebral ischemia/reperfusion injury in mice using chemical analysis and multiple biological assays. We found that both TPE and TPB increased antioxidant enzyme activities, suppressed OGD/R-induced ROS formation, decreased apoptosis, downregulated iNOS protein expression, and stimulated the Nrf2/HO-1 signaling pathway, which protected the brain against cerebral ischemia/reperfusion injury. Therefore, polyphenolic extracts of *T.* *chebula* may be a novel and promising strategy for ischemic stroke. 

There is an increasing interest in obtaining biologically and functionally natural plant-derived products that can be used to treat ischemic stroke. Recent studies have reported numerous remarkable properties of polyphenolic compounds [33]. *T. chebula* is rich in polyphenolic compounds. We isolated TPE and TPB from the methanol extract of *T. chebula*. Compared with vitamin C, TPE and TPB showed significantly stronger free radical scavenging abilities in vitro. Emerging evidence has demonstrated that therapies aimed at increasing antioxidant activity could alleviate brain injury in ischemic stroke by suppressing oxidative stress. 

Oxidative stress, which involves excessive generation of ROS, is crucially involved in ischemic stroke pathogenesis. Excessive ROS production causes neuronal dysfunction through multiple mechanisms, including apoptosis, inflammation and cellular necrosis [34]. Mitochondrial damage is strongly associated with the cell apoptosis process. There was a marked increase in the early apoptotic ratio and MMP in OGD/R-injured BV2 microglial cells. Moreover, TPE and TPB treatments considerably relieved OGD/R-induced cell apoptosis and mitochondrial dysfunction. This suggests that the polyphenolic extracts of *T. chebula* might protect BV2 microglial cells against OGD/R injury through mitochondria-induced apoptosis pathways. Similarly, another remarkable finding confirmed that TPE and TPB suppressed apoptosis in MCAO mice. Furthermore, ROS levels can be reduced by enzymatic antioxidants, for instance, SOD, glutathione peroxidase and catalase. Our results suggested that SOD and GSH-Px activities were increased in vitro. This abnormal increase in antioxidant activity might result from feedback regulation of excessive ROS production. Thus, we provided evidence that TPE and TPB treatments induced enhanced SOD activity after ischemia/reperfusion. In summary, polyphenolic extracts from *T. chebula* exerted neuroprotective effects by scavenging excessive ROS and improving the antioxidant systems.

As a critical nuclear transcription factor, Nrf2 is widely expressed and is an important antioxidant that maintains redox homeostasis in cells [18]. These studies suggest that Nrf2 plays a positive role against oxidative stress-induced brain damage [35]. Nrf2 pathway dysfunctions increase the brain infarct area and neuronal damage after ischemic stroke [36]. Furthermore, Nrf2 is a vital molecular target for the neuroprotective effects of numerous natural products by suppressing oxidative stress [37]. We provided evidence that Nrf2 protein expression was decreased after cerebral ischemia/reperfusion; however, TPE and TPB considerably increased Nrf2 protein expression. Immunofluorescence analysis revealed that TPE and TPB treatment increased Nrf2 translocation levels into the nucleus. In addition, TPE and TPB remarkably ameliorated the reduced expression of the binding protein Keap1 and the downstream antioxidant proteins HO-1 and GCS-γ after ischemia/reperfusion. Previous studies have shown that iNOS activation caused simultaneous nitric oxide production, which ultimately exacerbated oxidative stress damage [38]. We also found that TPE and TPB remarkably reduced excessive iNOS protein expression after ischemia/reperfusion. These results suggested that TPE and TPB exert neuroprotective effects against cerebral ischemia/reperfusion injury by activating the Nrf2/ HO-1 pathway and alleviating oxidative stress. 

In conclusion, TPE and TPB could be promising compounds with activity against cerebral ischemia/reperfusion induced oxidative damage, and they are potential agents for treating or preventing ischemic stroke. Moreover, this is the first report of the treatment of *T. chebula* extract in OGD/R cells and MCAO mice. Although we showed that TPE and TPB suppressed oxidative stress to prevent ischemia/reperfusion injury, we cannot say with certainty that protection of oxidative stress is the only reason. In addition, the compound from *T. chebula* extracts plays the main role in neuroprotective effects remains unclear, so there is a need for further studies to elucidate its underlying mechanism in clinical treatment.

## 4. Materials and Methods

### 4.1. Chemicals and Reagents

Methanol, ethyl acetate and n-butanol were purchased from Sinopharm Chemical Reagent Co., Wuhan, China. Dulbecco’s Modified Eagle’s Medium (DMEM) and fetal bovine serum (FBS) were obtained from Gibco BRL, NY, USA. Assay kits were purchased from Beyotime Biotechnology, Shanghai, China. We obtained anti-inducible nitric oxide synthase (iNOS), anti-HO-1, anti-GCS-γ, anti-Caspase3, anti-β-actin, and anti-LaminB antibodies from Absin Bioscience Inc., Shanghai, China. Anti-Keap1 and anti-Nrf2 antibodies were obtained from Boster Biological Technology Co., Wuhan, USA. Anti-Bax and anti-Bcl2 antibodies were purchased from Cell Signaling Technology Inc., MA, USA.

### 4.2. Plant Material and Preparation of Extracts

*T. chebula* was purchased from a local pharmacy, and medicinal materials were produced by Hubei Jingui Traditional Chinese Medicine Co., Wuhan, Ltd. (Production No. A170601). Subsequently, dried fruits were crushed into a coarse powder using a crusher and defatted using petroleum ether. Extraction was performed with 50% methanol followed by evaporation using a rotary evaporator (Yarong Biochemistry, Shanghai, China) to remove excess methanol. Subsequently, the concentrated solution was extracted with ethyl acetate solvent, followed by an n-butanol solvent; next, two solvent extracts were collected and concentrated separately. The concentrated solutions were adsorbed onto AD-8 macro porous resin; furthermore, water and ethanol were used to remove excess carbohydrates and low molecular weight compounds. Subsequently, 80% ethanol was used to elute the required polyphenols. Polyphenols of the ethyl acetate fraction from *T. chebula* extract (TPE) and n-butanol fraction (TPB) were concentrated and freeze-dried into powder in a vacuum freeze dryer (Yarong Biochemistry, Shanghai, China) [27,39].

### 4.3. Main Compounds and Antioxidant Activity In Vitro

Chemical composition analysis of TPE and TPB was performed using high-performance liquid chromatography (HPLC) with an ultraviolet (UV) detector. The compounds were separated using a PE C18 column. Mobile phases were acetonitrile and 1% acetic acid in water, respectively. The main ingredients, chebulinic acid and chebulagic acid, were detected at 280 nm wavelength [40].

To investigate the antioxidant capacities of TPE and TPB, 1, 1-diphenyl-2-picrylhydrazyl (DPPH•), 2-2-azino-di-(3-ethylbenzthiazoline sulfonic acid (ABTS•), and hydroxyl radicals (OH•) radical scavenging assays were performed as previously described [41]. The scavenging activity was assessed using the half effective inhibition concentration (IC50 value).

### 4.4. Cell Culture

BV2 microglial cells were purchased from Cell Bank (Shanghai, China) and cultivated in an incubator. In the OGD/R experiment, the culture medium was replaced with DMEM without glucose and FBS; furthermore, cells were cultivated in an anaerobic chamber (Billups-Rothenberg, Del Mar, CA, USA) containing 95% N2 and 5% CO_2_ for 6 h, and then transferred to a normal environment and medium for an additional 3 h [5].

TPE and TPB were completely dissolved in DMSO; additionally, the required concentrations were diluted with DMEM before use. BV2 microglial cells were exposed to OGD with or without TPE and TPB for 6 h and then placed in an aerobic environment in a complete medium for an additional 3 h. The different cell groups were harvested and used for subsequent experiments.

### 4.5. Measurement of Cell Viability and Enzyme Activities

Cell cytotoxicity and protection of TPE and TPB was measured by CCK-8 assay kit following the manufacturer’s specifications as a reference protocol. SOD, GSH-P x, MDA and ROS were measured using the kits following the specifications [42].

### 4.6. Detection of Apoptotic Ratio and Mitochondrial Membrane Potential

The apoptotic ratio was measured using Apoptosis Detection Kit. BV2 microglial cells from different groups were centrifuged and washed thrice using phosphate buffered saline (PBS), re-suspended in binding buffer, and incubated with working solution for 20 min in darkness. Fluorescence was recorded by a flow cytometer (BD Bioscience, San Jose, CA, USA).

JC-1 was seen as red-fluorescent aggregates in normal cells due to higher MMP. In contrast, JC-1 changed into a green-fluorescent monomer at low MMP due to external stimulation. This phenomenon can be applied to measure changes in MMP. BV2 microglial cells were centrifuged and incubated with JC-1. After washing thrice using PBS, cells were analyzed using a flow cytometer with ≥10,000 events per sample being recorded [43].

### 4.7. Middle Cerebral Artery Occlusion (MCAO) Model and Drug Treatment

C57BL/6 male mice were purchased from the Experimental Animal Center of Yangzhou University (Yangzhou, China; certificate number SCXK, 2017–0007). The experimental protocol was approved by the Ethic Committee of Animal Use for Teaching and Research, School of Medicine, Jianghan University. Mice were divided into four groups: sham, model, and model groups that received TPE (200 mg/kg) or TPB (200 mg/kg). After 1 week acclimatization, mice in treatment groups had intragastric administration of TPE or TPB at a dose of 200 mg/kg for 4 days, and mice in sham and model groups were administered an equal volume of saline. We performed the operation after the completion of intragastric administration on the fourth day.

Middle cerebral artery occlusion (MCAO) is currently the most common stroke model. The procedure was carried out as previously reported with minor modifications [44]. Briefly, after the mice were anesthetized, we isolated the carotid arteries, and then a monofilament nylon suture was introduced into internal carotid artery. After remaining for 2 h, the suture was removed from internal carotid artery for reperfusion (24 h). For mice in sham group, the procedure was similar except that the monofilament nylon suture was not inserted into internal carotid artery.

### 4.8. Western Blotting

Nuclear and cytoplasmic extraction kits were used to separate cytosolic and nuclear fractions of cells for subsequent Nrf2 determination. The total proteins were separated using 10% sodium dodecyl sulphate–polyacrylamide gel electrophoresis and transferred to 0.22-μm polyvinylidene fluoride membranes. Protein-bearing membranes were blocked and incubated overnight at 4 °C using antibodies against iNOS (1:500), HO-1 (1:1000), GCS-γ (1:1000), Keap1 (1:1000), Nrf2 (1:500), Bax (1:1000), Bcl2 (1:1000) and Caspase3 (1:1000). Additionally, the references of total proteins were performed using β-actin (1:3000) and the nuclear proteins were Lamin B (1:1000). Membranes were washed thrice using Tris-buffered saline and incubated with secondary antibodies. After washing thrice using TBST, immunoreactive protein bands were visualized and relative densitometric of the bands was measured using a chemiluminescence system (Gene Company, Hong Kong, China).

### 4.9. Immunofluorescence Staining

BV2 microglial cells were exposed to normal conditions or OGD/R with or without treatment using TPE and TPB. Subsequently, cells on glass sheets were collected into a glass container and fixed using cold acetone for 10 min, permeabilized using Triton X-100, blocked with BSA for 40 min and finally incubated with an anti-Iba1 (1:500) and an Nrf2 antibody (1:200) overnight at 4 °C. Thereafter, they were washed using TBST and incubated with the secondary conjugated antibodies for 1 h at 37 °C in the dark. After washing using TBST, cells were counterstained with Hoechst (1:1000) for 10 min. Images were captured using a fluorescence microscope (Olympus, Japan); furthermore, we visualized the localization of Iba1- or Nrf2-connected red fluorescence of the whole cell and the blue fluorescence of the nucleus [45].

### 4.10. Statistical Analysis

All experimental data were expressed as mean ± SD. One way ANOVA was applied to analysis of the data. Each experiment was repeated at least three times.

## Figures and Tables

**Table 1:** IC50 values of TPE and TPB in free radical scavenging experiments.

Substance	IC50 (μ g/mL)		
	DPPH•	ABTS•+	OH•
TPE	3.15 ± 0.02 **	58.71 ± 1.88 **	36.79 ± 2.44 **
TPB	3.48 ± 0.16 **	76.77 ± 1.67	253.16 ± 19.37 **
VC	5.52 ± 0.18	76.36 ± 2.33	2904 ± 97.97

Data are expressed as mean *±* SD; ** *p* < 0.01 vs. VC; TPE and TPB, vitamin C equivalent antioxidant capacity. TPB: polyphenols of *T. chebula* n-butanol extract; TPE: polyphenols of *T. chebula* ethyl acetate extract; VC: vitamin C.

## Data Availability

This study did not report any data.

## Sample Availability

Samples of the compounds are not available from the authors.

## Abbreviations

*T. chebula*, *Terminalia chebula* Retz; OGD/R, oxygen-glucose deprivation/reoxygenation; TPE, polyphenols of *T. chebula* ethyl acetate extract; TPB, polyphenols of *T. chebula* n-butanol extract; ROS, reactive oxygen species; MMP, mitochondrial membrane potential; Nrf2, nuclear transcription factor erythroid-2 related factor 2; HO-1, heme oxygenase-1; SOD, superoxide dismutase; GSH, glutathione peroxidase; MCAO, middle cerebral artery occlusion; iNOS, inducible nitric oxide synthase; HPLC, high-performance liquid chromatography; FITC, fluorescein isothiocyanate; PI, propidium iodide; MDA, malondialdehyde; GSH-P x, glutathione peroxidase; GCS-γ, gamma glutamylcysteine synthetase; Keap1, kelch ECH-associated protein 1; DMEM, Dulbecco′s Modified Eagle′s Medium; FBS, fetal bovine serum; DMSO, dimethyl sulfoxide; PBS, phosphate buffered saline.
